# Intermittent peripheral exposure to lipopolysaccharide induces exploratory behavior in mice and regulates brain glial activity in obese mice

**DOI:** 10.1186/s12974-020-01837-x

**Published:** 2020-05-25

**Authors:** Hui-Ting Huang, Po-See Chen, Yu-Min Kuo, Shun-Fen Tzeng

**Affiliations:** 1 Department of Life Sciences, College of Bioscience and Biotechnology, National Cheng Kung University, Tainan, Taiwan; 2 Department of Psychiatry, College of Medicine, National Cheng Kung University, Tainan, Taiwan; 3 Institute of Basic Medical Sciences, Department of Cell Biology and Anatomy, College of Medicine, National Cheng Kung University, Tainan, Taiwan; 4 Department of Life Sciences, National Cheng Kung University, #1 University Road, Tainan, Taiwan

**Keywords:** Peripheral inflammation, Obesity, Microglia, Gliosis, Exploration

## Abstract

**Background:**

Consecutive peripheral immune challenges can modulate the responses of brain resident microglia to stimuli. High-fat diet (HFD) intake has been reported to stimulate the activation of astrocytes and microglia in the arcuate nucleus (ARC) of the hypothalamus in obese rodents and humans. However, it is unknown whether intermittent exposure to additional peripheral immune challenge can modify HFD-induced hypothalamic glial activation in obese individuals.

**Methods:**

In this study, we administered 1 mg/kg LPS (or saline) by intraperitoneal (i.p.) injection to 8-week-old male mice after 1, 2, or 8 weeks of a regular diet (show) or HFD. The level of interleukin-1β (IL-1β) and tumor necrosis factor-α (TNF-α) expression in the plasma and hypothalamic tissue was analyzed 24 h after each LPS injection. The behaviors of the animals in the four groups (the chow-saline, chow-LPS, HFD-saline, and HFD-LPS groups) were examined 5 months after exposure to chow or a HFD. Morphological examination of microglia in related brain regions was also conducted.

**Results:**

The plasma levels and hypothalamic mRNA levels of IL-1β and TNF-α were significantly upregulated 24 h after the first injection of LPS but not after the second or third injection of LPS. Chow-LPS mice displayed increased exploratory behavior 5 months after feeding. However, this LPS-induced abnormal exploratory behavior was inhibited in HFD-fed mice. Chronic HFD feeding for 5 months induced apparent increases in the number and cell body size of microglia, mainly in the ARC, and also increased the size of microglia in the nucleus accumbens (NAc) and insula. Moreover, microglial activation in the ARC, anterior cingulate cortex (ACC), insula, and basolateral amygdala (BLA) was observed in chow-LPS mice. However, microglial activation in the analyzed brain regions was suppressed in HFD-LPS mice.

**Conclusions:**

Altogether, the results indicate that intermittent peripheral challenge with LPS might prime microglia in the ARC and NAc to modify their response to chronic HFD feeding. Alternatively, chronic HFD feeding might mediate microglia in LPS-affected brain regions and subsequently suppress LPS-induced atypical exploratory behavior. Our findings suggest that the interaction of intermittent acute peripheral immune challenges with chronic HFD intake can drive microglia to amend the microenvironment and further modify animal behaviors in the later life.

## Background

Microglia, resident macrophages of the central nervous system (CNS), participate in synaptic pruning and microenvironmental homeostasis during CNS development [1]. Microglial activation is associated with neuroinflammation in human brain neurological diseases (such as Alzheimer’s disease and Parkinson’s disease), traumatic CNS injuries (such as stroke and spinal cord injury), and neuropsychiatric disorders [2]. Microglial activation and increased production of inflammatory cytokines in response to stress are believed to be important triggers for depression in humans and in animal models [3]. Peripheral inflammation has been demonstrated to be a trigger for microglial activation and sickness behavior in humans and animals [4, 5]. In addition, astrocytes, abundant glial cells that constitute approximately 40% of brain cells, participate in homeostasis in the healthy CNS and neuropathogenesis induced by CNS injury or peripheral inflammation [6, 7]. The two glial cell types are also involved in metabolic disorder-linked neuroinflammation [8].

Systemic inflammation induced by peripheral administration of a bolus of LPS (1 mg/kg) significantly induces acute microglial activation, mainly in the cortex, caudate putamen, and substantia nigra, in mice [9]. In addition, chronic systemic low-grade inflammation is a common feature of obesity that can be caused by an imbalance in energy metabolism and irregular lifestyle [10–12]. Obesity-induced central neuroinflammation is also involved in the disruption of hypothalamic homeostasis [13]. The accumulation of microglia in the hypothalamic ARC is also a common feature of obese mice [12–16]. In addition, endotoxemia, a phenomenon in which alterations in gut permeability increase plasma LPS and activate macrophages, has been found in high-fat diet (HFD)-fed animals [17]. Diet-induced obesity in a rat model enhances sickness symptoms triggered by a single i.p. injection of LPS and delays recovery from the LPS-induced impairment of social interaction at 30 h post-injection [18]. Moreover, acute immune challenge by i.p. injection of LPS in obese rats can cause an increase in proinflammatory mediators (TNFα, IL-1β, IL6, IκBα, and COX2) in the hypothalamus [19], suggesting that the enhancement of inflammation can be induced by obesity combined with immune challenge. On the other hand, the findings have demonstrated that repeated acute immune challenges using a low dose of LPS for four sequential days can induce long-term alterations of brain immune responses [20]. However, the impact of repeated acute inflammatory challenges on the response of microglia to HFD feeding is unknown.

Systemic infection that can cause neurodegeneration and affective behaviors at the later-life time points have been documented [21, 22]. Considering that individuals could meet acute infection time to time in their life time, this study aimed to examine whether the acute systemic inflammation induced by intermittent immune challenges can cause long-last effect on the brain microglia of obese mice. We hypothesized that hypothalamic microglia activation can be modified by the administration of a high dose of LPS (1 mg/kg) at the three early time points during the HFD feeding period through a peripheral routine. We also determined whether behavioral abnormalities developed in obese mice under the influence of intermittent LPS administration. As unexpected, LPS challenges caused the development of an atypical exploratory behavior in chow-fed mice at the later time point, whereas HFD feeding for 5 months suppressed this LPS-induced explorative behavior. Moreover, HFD-induced microglial activation in the ARC and NAc was attenuated in obese mice that received intermittent peripheral LPS challenges. Overall, this study provides important evidence that the interplay between intermittent LPS challenge and chronic HFD feeding can amend their individual effects on microglial activation in distinct brain regions and possibly alter LPS-induced exploratory behavior.

## Materials and methods

### Animals

**Fig. 1:**
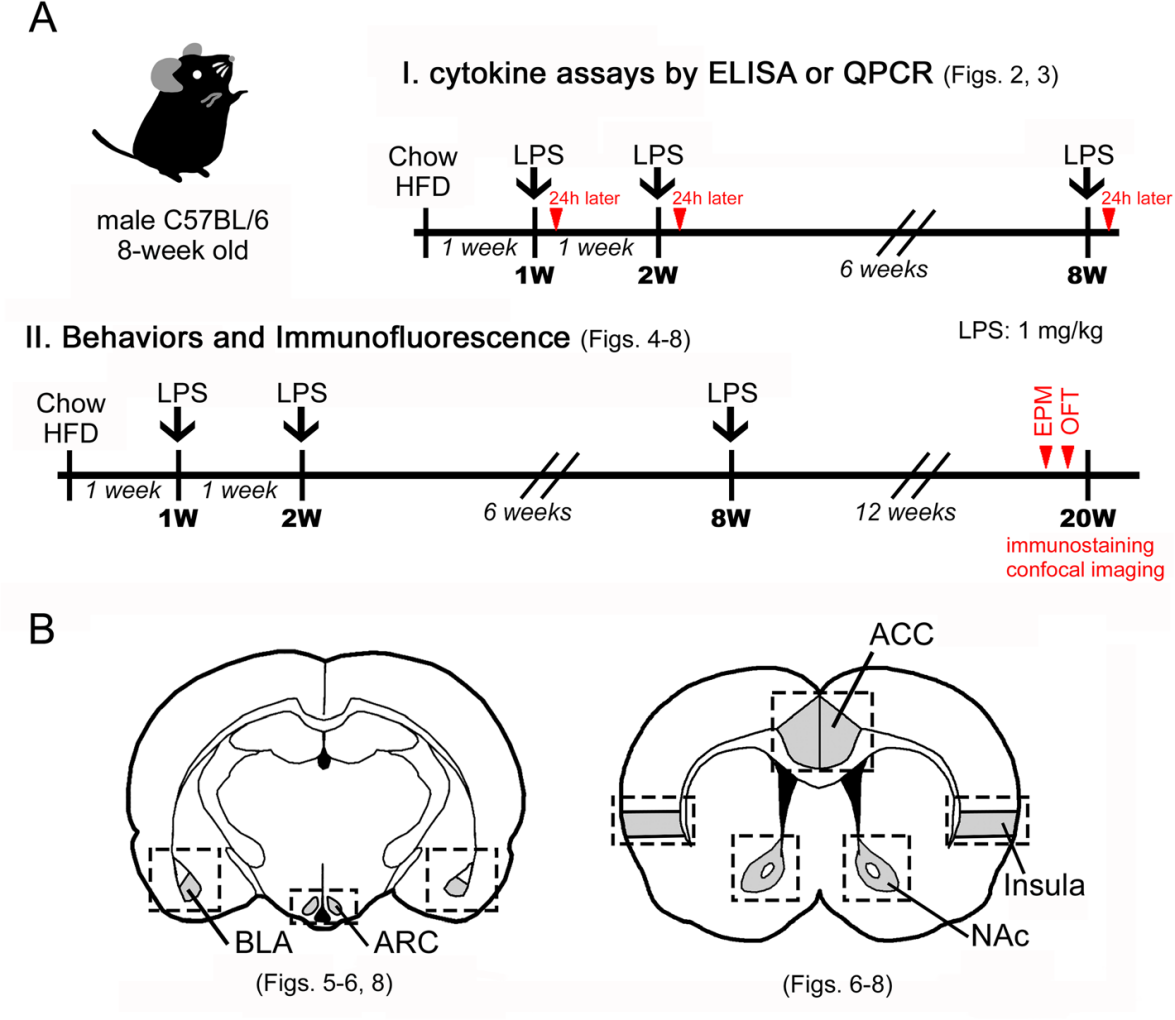
The schematic diagram illustrates the experimental design (**a**) and brain regions (**b**) used for Iba1 and GFAP immunofluorescence (IF). In brief, mice fed chow or a HFD for 1 week (1 w), 2 weeks (2 w), or 8 weeks (8 w) received 1 mg/kg LPS intraperitoneally. Plasma and hypothalamic tissues were prepared 24 h after each LPS injection to measure TNF-α and IL-1β via QPCR. Alternatively, mice received an injection of 1 mg/kg LPS at the three indicated time points (1 w, 2 w, 8 w) during chow or HFD feeding and then continued to be fed chow or a HFD for up to 5 months (20 w). Animal behavior was analyzed using the EPM and OFT before the animals were sacrificed. Brain sections were collected to examine Iba1^+^ microglia and GFAP^+^ astrocytes in the indicated brain regions (the ARC, BLA, NAc, ACC, and insula)

Male C57BL/6 mice (8 weeks old, 20.39 ± 0.21 g) were purchased from the National Cheng Kung University Laboratory Animal Center. All mice were placed in individual cages (2 animals per cage) and housed under standard room conditions (room temperature: 23 ± 2 °C; humidity: 58 ± 2%; 12-h light/dark cycle). The mice were fed a normal diet (Laboratory Rodent Diet #5001; LabDiet, St. Louis, MO, USA) or HFD (Rodent Purified Diet #58Y1; TestDiet, St. Louis, MO, USA) ad libitum. Lipopolysaccharide (LPS; 1 mg/kg; O55:B5, Sigma, St Louis, MO; Cat# L6529) was administered by i.p. injection 1 week, 2 weeks, and 8 weeks after HFD feeding. Accordingly, the animals were randomly divided into 4 groups. The chow- and HFD-fed animals were injected with saline (chow-saline; HFD-saline) or LPS (chow-LPS; HFD-LPS) at the three indicated time points. The animals used to measure plasma cytokine levels and hypothalamic cytokine gene expression were sacrificed 24 h after the third injection of either saline or LPS (Fig. 1a (I)). For behavioral tests and immunofluorescence, the animals were fed chow or a HFD for the entire 5-month feeding period (Fig. 1a (II)). The animals were sacrificed by i.p. injection with Zoletil 50 (diluted 5× in saline; 0.05–0.06 mL/10 g; Virbac Taiwan Co., Ltd., Taipei, Taiwan), which was recommended by the university animal center because it can effectively reduce pain in animals. The use of the animals followed the National Institutes of Health (NIH) Guidelines for Animal Research (Guide for the Care and Use of Laboratory Animals) and was approved by the National Cheng Kung University Institutional Animal Care and Use Committee, Tainan, Taiwan (IACUC approval number: 106060).

### Immunofluorescence

Brain tissues were fixed with 4% paraformaldehyde overnight and then transferred to 30% (w/v) sucrose in PBS. The tissues were mounted in Tissue Tek optimal cutting temperature compound (Electron Microscopy Sciences, Torrance, CA, USA) and sectioned at a thickness of 20 μm. Floating coronal brain sections were treated with 1% Triton-X-100 in PBS at 4 °C overnight and then incubated in an anti-ionized calcium binding adaptor molecule 1 (Iba1) antibody (for microglia; Wako, Cat# 019-19741, RRID:AB_839504) or an anti-glial fibrillary acidic protein (GFAP) antibody (for astrocytes; Millipore, Cat# AB5804, RRID:AB_2109645) in PBS containing 0.1% Triton X-100 and 1% horse serum at 4 °C overnight. The tissues were subsequently incubated with biotinylated secondary antibodies for 1 h and then incubated with Alexa 488/Cy3-avidin (1:200) for 45 min. An Olympus FLUOVIEW FV1000 confocal laser scanning microscope (FV1000, Tokyo, Japan) was used to observe the stained tissue via 405, 488, or 594 nm lasers.

### Animal behavior tests

The assays were performed according to previously described protocols [23]. Video tracking software (Ethovision, Noldus, Netherlands) was used for automatic recording and analysis of animal behavior. The procedures are described briefly below.

#### Elevated plus maze

The elevated plus-maze (EPM) contained a center zone, two open arms (length 30 cm, width 5 cm), and two closed arms (length 30 cm, width 5 cm). The platform of the maze was elevated to 80 cm above the ground. Animal behavior was recorded with a video camera positioned above the maze. All animals were placed in a silent, dark room with dim red light for 30 min before the test. Behavior was recorded for 5 min before each mouse was placed in the central zone and facing the open arm. The time spent in the open arms and total movement (m) were analyzed.

#### Open field test

The open field test (OFT) was performed in a white plastic box (55 cm × 55 cm × 33 cm). All animals were placed in a silent, dark room with dim red light for 30 min before the test. Each mouse was placed in the central zone of the box, and behavior was recorded for 5 min. The time spent in the central zone and total movement (m) were analyzed.

### Measurement of microglial number and cell body size

Iba1^+^ microglia in different brain regions (the ARC, NAc, BLA, ACC, and insula) were examined by counting the number of microglia and quantifying the size of microglial cell bodies using NIH ImageJ analysis software as previously described [12]. In brief, four randomly selected images per brain section were merged and captured in multiple 1-μm-thick steps using an Olympus FLUOVIEW FV1000 confocal laser scanning microscope. Four brain sections containing different brain regions were collected from three animals from each group and quantified.

### Quantitative real-time polymerase chain reaction

The animals were sacrificed and perfused with 0.9% saline prepared in diethylpyrocarbonate water (Sigma; Cat# D5758). The hypothalamus was removed and lysed with TRIzol™ (Invitrogen; Cat# 15596018) for RNA extraction. One microgram of RNA was reacted with MMLV reverse transcriptase (Invitrogen; Cat# 28025-013) to generate cDNA. PCR amplification was performed using specific primers and Fast SYBR® Green Master Mix (Applied Biosystems; Cat# 4385612); the PCR conditions were 95 °C for 10 min followed by 40 cycles of 95 °C for 10 s, annealing at 65 °C for 10 s, and extension at 72 °C for 2 s. The primers used in this study were designed using Primer-BLAST software provided by the National Center for Biotechnology Information and synthesized by Genomics (Taipei, Taiwan). Cyclophilin A (CyPA) was used as an internal control. StepOne Software v2.1 (Applied Biosystems) was used to determine the cycle-threshold fluorescence values. The expression level of the target genes relative to that of the internal control was presented as 2^−ΔCT^, where ΔCT = (Ct_target gene_ − Ct_CyPA_). The sequences of the specific primers for IL-1β, TNF-α, and CyPA are as follows: mouse IL1β (NM_008361.4) forward: 5′-TGCCACCTTTTGACAGTGATGA-3′, reverse: 5′-AAGGTCCACGGGAAAGACAC-3′; mouse TNF-α (NM_008361.4) forward: 5′-CCGGACTCCGCAAAGTCTAA-3′, reverse: 5′-ACCGTCAGCCGATTTGCTAT-3′; and mouse CyPA (NM_008907.1) forward: 5′-CGTCTGCTTCGAGCTGTTTG-3′, reverse: 5′-GTAAAATGCCCGCAAGTCAA-3′.

### Measurement of plasma TNF-α and IL-1β levels

After the animals were anesthetized with Zoletil 50 (diluted 5× in saline, 0.05–0.06 ml/10 g), blood (1 ml per animal) was collected via cardiac puncture using a 26G needle rinsed with 10 μl of heparin (5000 IU/mL; Leo Pharmaceutical, Ltd., Denmark) and centrifuged at 3000×*g* for 10 min. TNF-α and IL-1β levels in the plasma samples were analyzed using commercial quantification enzyme-linked immunosorbent assay (ELISA) kits. The protocol provided by the vendor was followed (TNF-α: R&D, Cat# MTA00B; IL-1β: R&D, Cat# MLB00C).

### Statistical analysis

The significance of differences between the groups (the chow-saline, chow-LPS, HFD-saline, and HFD-LPS groups) at the different time points observed in this study was determined using one-way ANOVA with Sidak’s post hoc test. Each value is expressed as the mean ± SEM of three animals per group at different time points (TNF-α and IL-1β expression) or 12 tissue sections of a specific brain region from 3 animals (Iba1 immunofluorescence).

## Results

### Hypothalamic inflammation after LPS administration

**Fig. 2:**
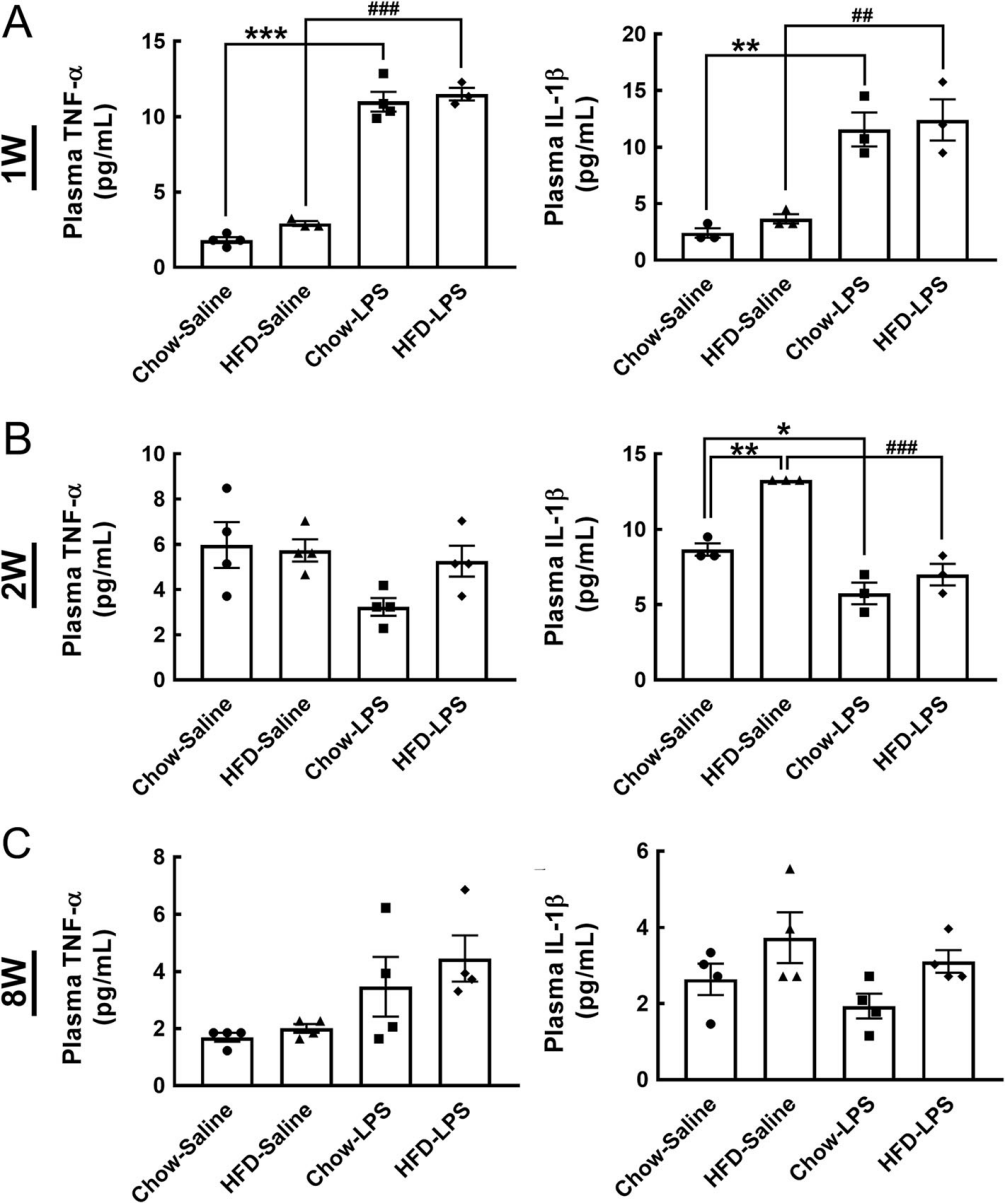
The plasma levels of TNF-α and IL-1β were increased by LPS injection into mice fed by chow or HFD. As illustrated in Fig. 1a, blood samples were collected from animals from the four groups 24 h after saline or LPS injection at 1 week (**a**), 2 weeks (**b**), and 8 weeks (**c**). The plasma preparation method was described in the “Materials and methods” section, and the samples were subjected to TNF-α and IL-1β ELISA assays. The data are presented as the mean ± SEM (*n* = 3–4 animals in each group). *p < 0.05, **p < 0.01, ***p < 0.001 versus the chow-saline group; ^##^*p* < 0.01, ^###^*p* < 0.001 versus the HFD-saline group

**Fig. 3:**
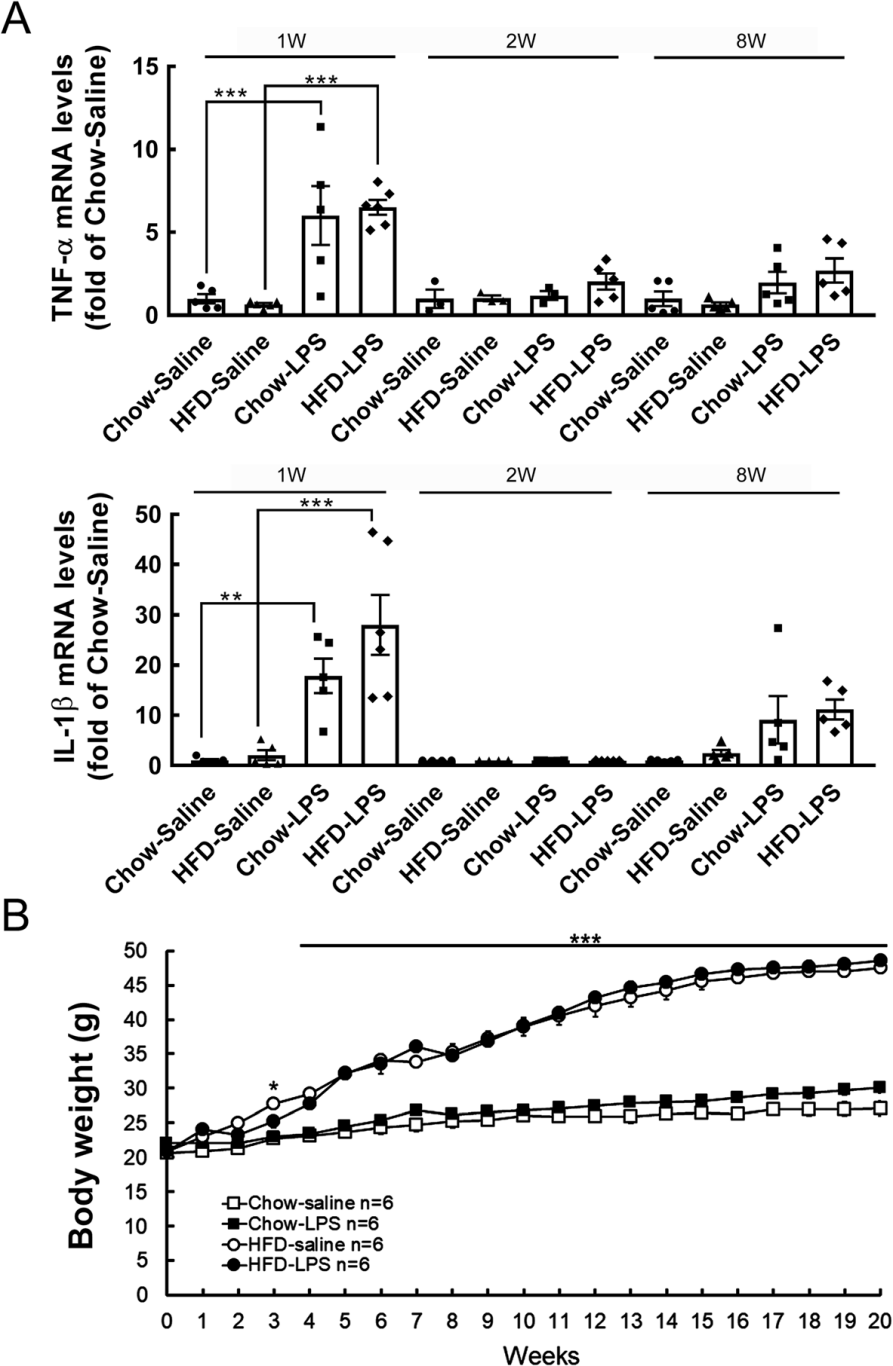
Hypothalamic TNF-α and IL-1β levels were increased after the first LPS injection. **a** As illustrated in Fig. 1a, hypothalamic tissues were collected from animals from the four groups (the chow-saline, HFD-saline, chow-LPS, and HFD-LPS groups) 24 h following saline or LPS injection (1 w, 2 w, and 8 w) and then subjected to QPCR to measure TNF-α and IL-β mRNA expression. The data are presented as the mean ± SEM (*n* = 3–6 animals in each group). **p < 0.01, ***p < 0.001 versus the chow and HFD groups. **b** The body weight of animals from the four animal was measured weekly for 5 months. The data are presented as the mean ± SEM (*n* = 6 animals in each group). *p < 0.05, ** *p* < 0.01, ***p < 0.001 versus the chow group

The declined expression of proinflammatory cytokines (TNF-α and IL-1β) in the hypothalamus at 1 week and 2 weeks post-HFD feeding has been reported [14, 16]. Previously, we have shown that IL-1β was increased in the hypothalamus at 2 months (8 weeks) after HFD feeding [12]. Thus, to determine whether intermittent systemic inflammation intensified hypothalamic inflammation in HFD-fed mice, we peripherally administered a high dose of LPS (1 mg/kg) at three time points (1 week, 2 weeks, and 8 weeks) during the early HFD administration period (Fig. 1a). The expression of proinflammatory cytokines (TNF-α and IL-1β) in the hypothalamus was examined 24 h after each LPS injection using QPCR analysis (Fig. 1a). As shown in Fig. 2a, the protein levels of TNF-α and IL-1β in the plasma were increased 24 h after the first LPS injection in chow- and HFD-fed mice. Similarly, compared to that detected in the chow-saline and HFD-saline groups, the mRNA expression of TNF-α and IL-1β in the hypothalamus of chow- and HFD-fed mice was significantly upregulated by the first LPS injection (Fig. 3a). However, the plasma level of TNF-α in HFD-fed mice was not affected, and its level in chow-fed mice showed a decreasing trend 24 h after the second LPS injection (Fig. 2b). Note that an increase in plasma IL-1β protein was detected in mice after HFD feeding for 2 weeks. Interestingly, the second LPS injection resulted in a reduction in the plasma level of IL-1β in chow- and HFD-fed mice when compared to the relative control group (Fig. 2a). Nevertheless, a non-significant difference in the expression of the two genes in the hypothalamus of animals from the four groups was observed after the second LPS injection (Fig. 3a). The plasma levels and gene expression of TNF-α and IL-1β in the hypothalamus were not significantly different from those detected in the chow-saline and HFD-saline groups (Fig. 2c and Fig. 3a). Despite that microglia accumulation in the hypothalamic ARC region was evident at 24 h after each LPS injection into chow- or HFD-fed mice (Fig. S1), we noticed that the three intermittent injections of LPS caused no change in the body weight of mice continuously fed either chow or a HFD for 5 months (Fig. 3b). Moreover, LPS administrations had no effect on food intake in HFD-fed mice, although it did reduce food intake in the chow-fed group (Fig. S2). Overall, the results demonstrate that the three intermittent peripheral injections of LPS applied at early time points during HFD feeding may have primed the response of immune cells and hypothalamic cells to chronic HFD feeding.

### Chronic HFD feeding attenuates LPS administration-induced exploratory behavior in mice

**Fig. 4:**
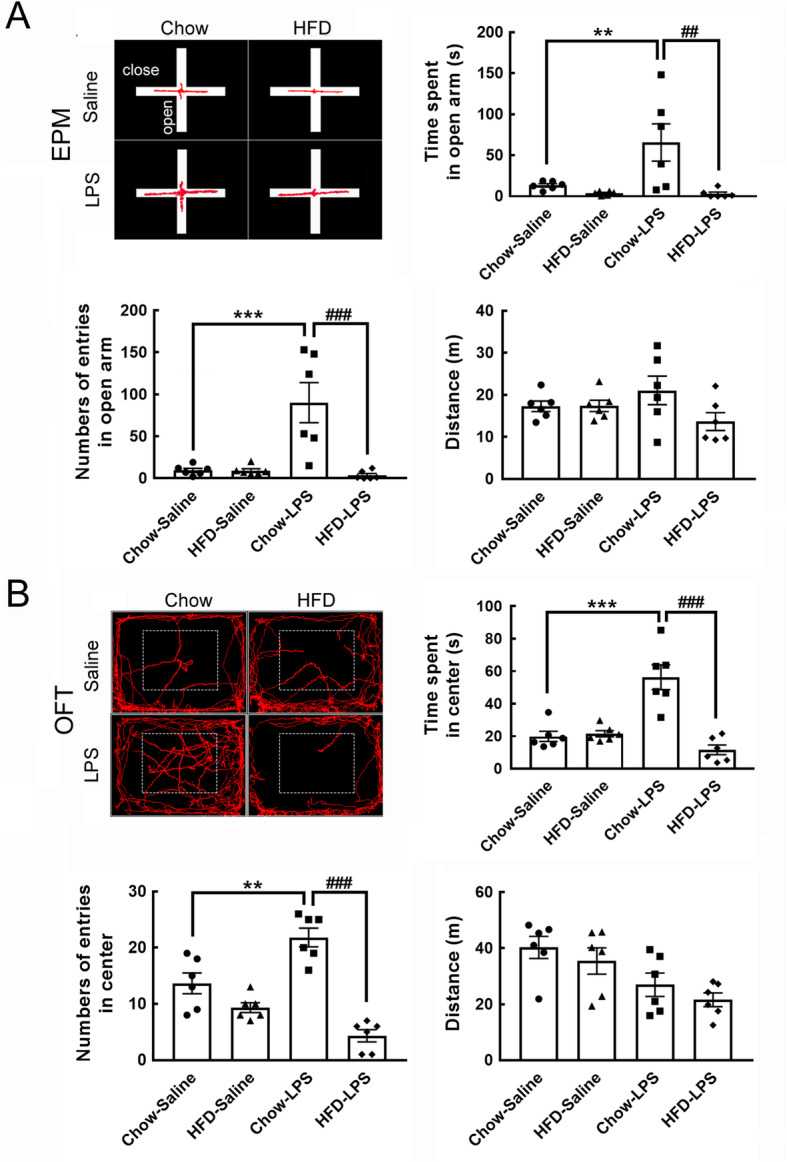
Chronic HFD feeding attenuated the suppression of LPS-induced behavior in mice. **a** Representative images from EPM analysis showing the walking track of animals from the four groups (left panel). The time spent in the open arms (seconds), number of entries into the open arms, and total distance traveled were measured. **b** The representative images indicate the walking tracks of animals from the four groups in the OFT (left panel). The time spent in the center of the OFT (seconds), number of entries into the center of the OFT, and total walking distance were measured. The data are presented as the mean + SEM. The data shown in **a** and **b** are presented as the mean + SEM of 6 animals from each group **a**, **b**. **p < 0.01, ***p < 0.001 versus the chow-LPS group; ^###^*p* < 0.001 versus the HFD-LPS group

Given that systemic inflammation induced by a bolus injection of LPS can evoke mice to develop anxious and depressive behavior later [24, 25], the behaviors of animals from the four groups were analyzed using the EPM and OFT after approximately 5 months of HFD feeding (Fig. 1a). The animals in the chow-saline and HFD-saline groups spent similar amounts of time in the open arms and showed no difference in the number of entries into the open arm (Fig. 4a). Surprisingly, intermittent LPS administration increased the time spent in and number of entries into the open arms of the chow-fed group (chow-LPS), whereas this behavior was significantly suppressed in HFD-LPS mice. No differences in the time spent in the closed arms or the number of closed-arm entries were observed between the four animal groups (Fig. S3). The results of the other behavioral assay, the OFT, also indicated that compared to animals from the other three groups, animals from the chow-LPS group spent more time in the center and exhibited an increased number of entries into the center (Fig. 4b). These observations reveal that the three intermittent injections of LPS at early time points induced chow-fed animals to develop intensive exploratory-like behavior at a later time point (i.e., 5 months in this study), whereas chronic HFD feeding suppressed LPS-induced exploratory behavior in mice.

### Intermittent LPS injections mediate ARC microglia in response to chronic HFD feeding

**Fig. 5:**
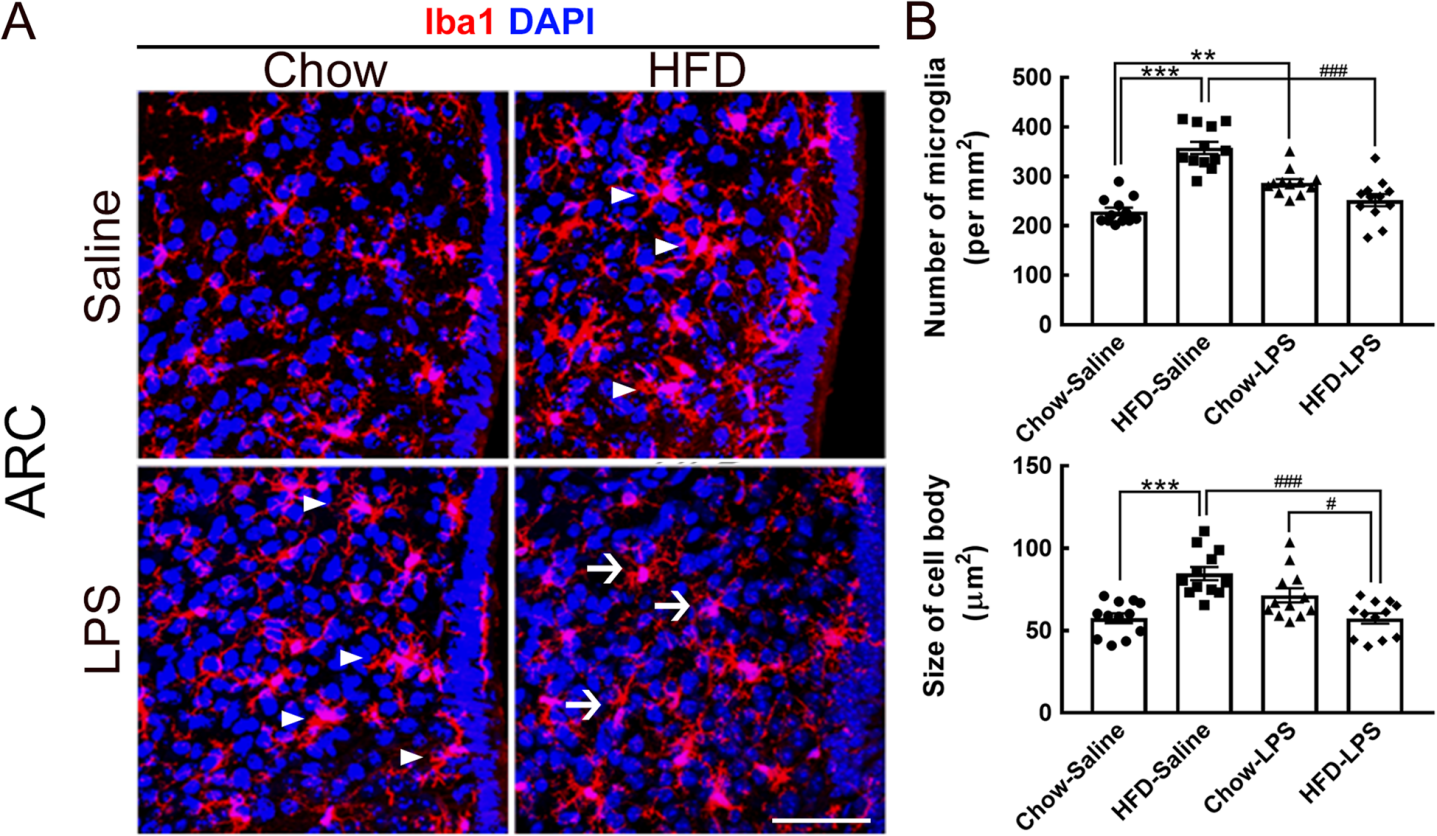
Morphological alteration of microglia in the hypothalamic arcuate nucleus was induced by intermittent LPS administration into HFD-fed mice. **a** Brain tissue sections containing the hypothalamic arcuate nucleus (ARC) were prepared from animals from the four groups after feeding for 5 months and then subjected to Iba1 immunofluorescence (red) and DAPI nuclear counterstaining (blue). Representative active microglia with hypertrophic shapes are indicated by arrowheads. Arrows indicate microglia with a small size and fine processes in the HFD-LPS group. **b** The number of Iba1^+^ microglia accumulated in the ARC (per mm^2^) in the four groups was quantified. In addition, the average cell body size of ARC microglia was measured. The data are presented as the mean ± SEM (*n* = 12 tissue sections from 3 animals from each group). **p < 0.01, ***p < 0.001 versus the chow-saline group; ^#^*p* < 0.05, ^###^*p* < 0.001 versus the HFD-LPS group. Scale bar in *A* = 50 μm

We previously reported that HFD feeding for 2, 3, and 4 months prolongs the accumulation of Iba1^+^ microglia with activated shapes in the ARC [12]. Here, the results showed that hypertrophic microglia were continuously observed in the ARC when mice were fed a HFD (the HFD-saline group) for up to 5 months (Fig. 5a, arrowheads). LPS administration at early time points caused an increase in the number and cell body size of ARC microglia in chow-fed mice compared to mice in the chow-saline group (Fig. 5a, arrowheads; Fig. 5b). However, the number and cell size of microglia in the chow-LPS group tended to be decreased compared with those observed in the HFD-saline group (Fig. 5b). Moreover, intermittent LPS injections significantly reduced the number of ARC microglia in HFD-fed mice compared to mice in the HFD-saline and chow-LPS groups (Fig. 5b). Moreover, ARC microglia in the HFD-LPS group displayed reduced cell body sizes (Fig. 5a, arrows). These findings indicate that intermittent LPS administration at early time points might modulate the sensitivity of ARC microglia to persistent HFD feeding.

### Suppression of microglial activation in different brain regions by intermittent LPS injections combined with chronic HFD feeding

**Fig. 6:**
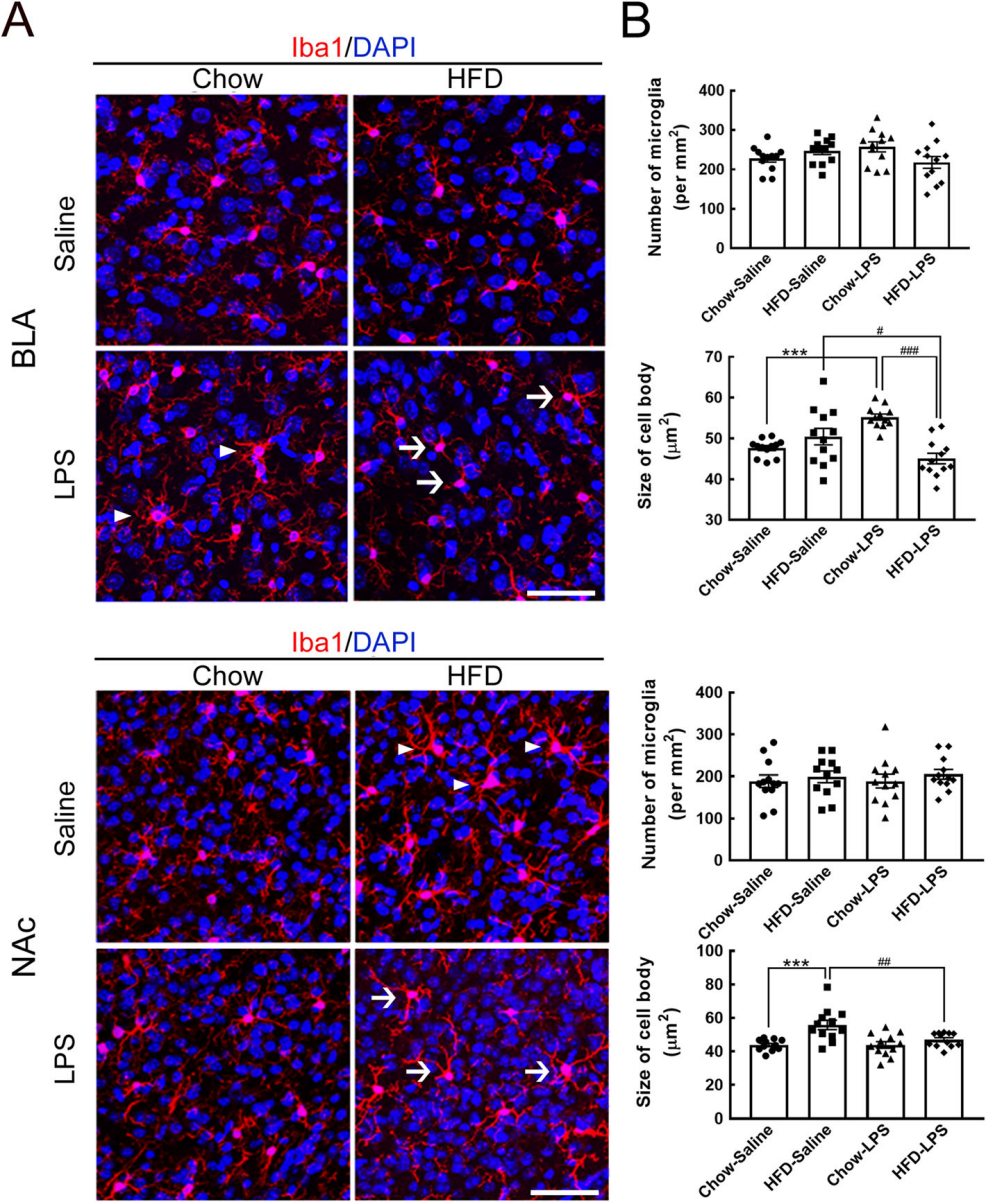
Microglial activation in the BLA and NAc was attenuated by intermittent LPS administration and chronic HFD feeding. **a** Brain tissue sections containing the BLA or NAc were prepared from animals from the four groups after feeding for 5 months (Fig. 1a) and then subjected to Iba1 immunofluorescence (red) and DAPI nuclear counterstaining (blue). Representative active microglia with hypertrophic shapes are indicated by arrowheads. Arrows indicate microglia with a small size and fine processes in the HFD-LPS group. **b** The number of Iba1^+^ microglia accumulated in the BLA or NAc (per mm^2^) in the four groups was quantified. In addition, the average cell body size of microglia in the two regions was measured. The data are presented as the mean ± SEM (*n* = 12 tissue sections from 3 animals from each group). ***p < 0.001 versus the chow-saline group; ^#^*p* < 0.05, ^##^*p* < 0.01, ^###^*p* < 0.001 versus the HFD-LPS group. Scale bar in *A* = 50 μm

Since LPS administration can induce microglial activation in emotion-associated brain regions, such as the basolateral amygdala (BLA) and nucleus accumbens (NAc) [9], microglia in the two brain regions collected from the four animal groups (i.e., the chow-saline, HFD-saline, chow-LPS, and HFD-LPS groups) was examined at 5 months after HFD feeding (Fig. 1). As shown in Fig. 6a (arrowheads), early LPS administration in chow-fed mice increased cell size in the BLA, although the number of BLA microglia was not increased in chow-fed mice (Fig. 6b). However, the cell body size of BLA microglia was significantly reduced in the HFD-LPS group (Fig. 6a, arrows) compared to the chow-LPS and HFD-saline groups (Fig. 6b). As in the BLA, early LPS administration and chronic HFD feeding did not change the level of microglial activation in the NAc (Fig. 6a). However, NAc microglia were found to exhibit a larger cell size in HFD-fed mice than in mice from the chow-saline, chow-LPS, and HFD-LPS groups (Fig. 6b). The results reveal that the interplay of peripherally injected LPS with chronic HFD feeding suppressed the activity of microglia in the BLA and NAc.

**Fig. 7:**
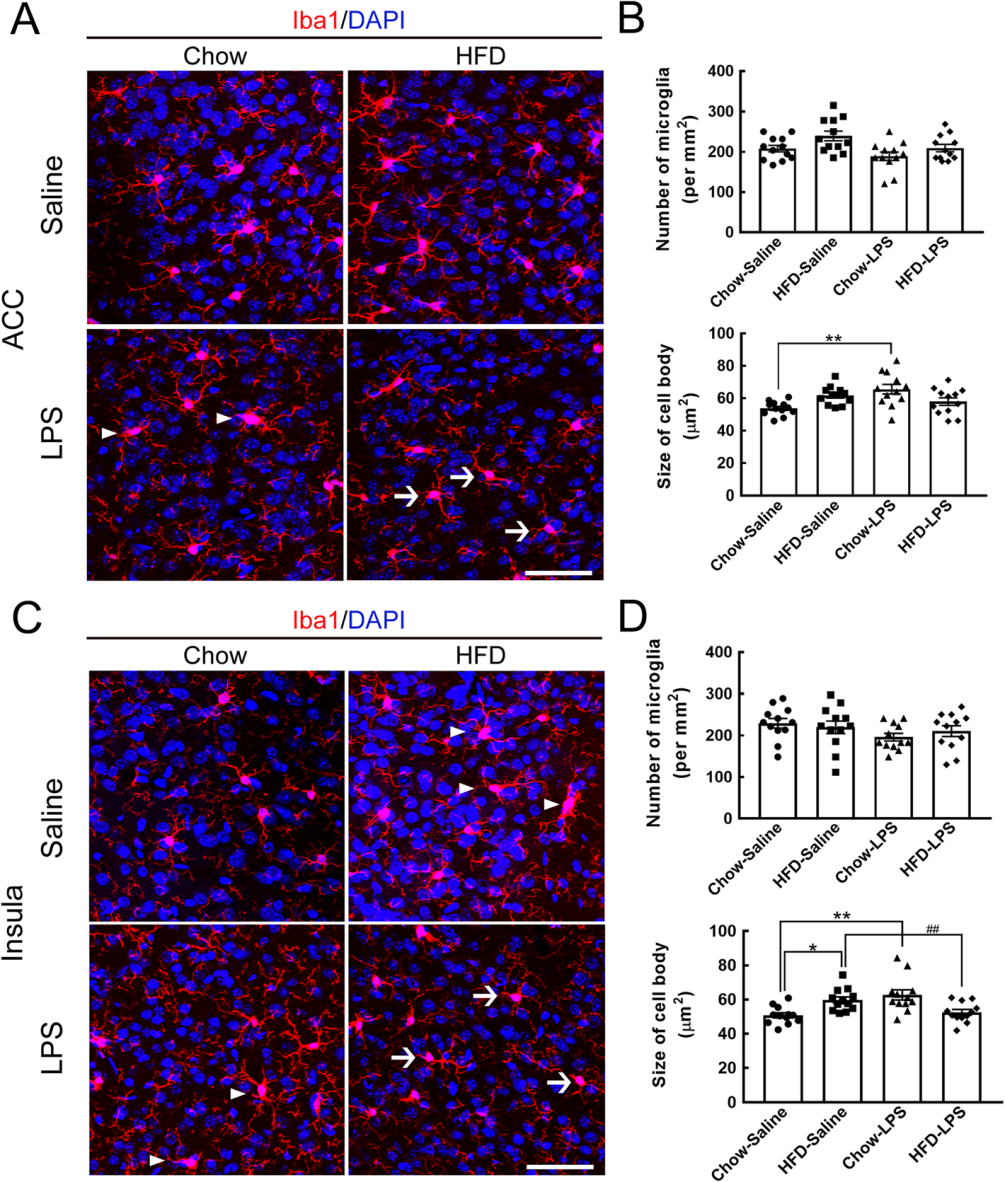
Microglial activation in the ACC and insula was attenuated by intermittent LPS administration and chronic HFD feeding. **a** Brain tissue sections containing the ACC or insula were prepared from animals from the four groups after feeding for 5 months (Fig. 1a) and then subjected to Iba1 immunofluorescence (red) and DAPI nuclear counterstaining (blue). Active microglia with hypertrophic shapes are indicated by arrowheads in representative confocal images. Arrows point to microglia with a small size and fine processes in the HFD-LPS group. **b** The number of Iba1^+^ microglia accumulated in the ACC or insula (per mm^2^) in the four groups was quantified. In addition, the average cell body size of microglia in the two regions was measured. The data are presented as the mean ± SEM (*n* = 12 tissue sections from 3 animals from each group). *p < 0.05, **p < 0.01 versus the chow-saline group; ^##^*p* < 0.01 versus the HFD-LPS group. Scale bar in *A* = 50 μm

Furthermore, we investigated whether early LPS administration affected microglial activation in emotion-associated cortical areas, specifically the anterior cingulate cortex (ACC) and the insula, in chow- and HFD-fed mice (Fig. 1). Although no change in the number of ACC microglia was induced in chow- or HFD-fed mice by early LPS administration, the body size of ACC microglia was increased in chow-fed mice that received early LPS administration (Fig. 7a, arrowheads; Fig. 7b). Although most ACC microglia in the HFD-fed mice that received early LPS administration were small (Fig. 7a, arrows), there was no significant difference compared to the chow-LPS group (Fig. 7b). Microglia in the insula were examined, and it was found that the microglial cell size was increased in the insula of the chow-LPS and HFD-saline groups (Fig. 7c, arrowheads; Fig. 7d); however, the number of microglia in the insula was not changed in either of the two animal groups. However, a reduction in the cell size of microglia in the insula was observed in the HFD-LPS group compared to the chow-LPS and HFD-saline groups (Fig. 7c, arrows; Fig. 7d). The data suggest that although microglial activation in emotion-associated brain regions was induced by either chronic HFD feeding or early LPS administration, the combination of early LPS administration and chronic HFD feeding did not excite microglia, suggesting that LPS administration might prime microglia at early time points to regulate their activity under the influence of chronic HFD feeding.

### Enhancement of the elaboration of astrocytic processes in the ARC and BLA by early LPS administration and chronic HFD feeding

**Fig. 8:**
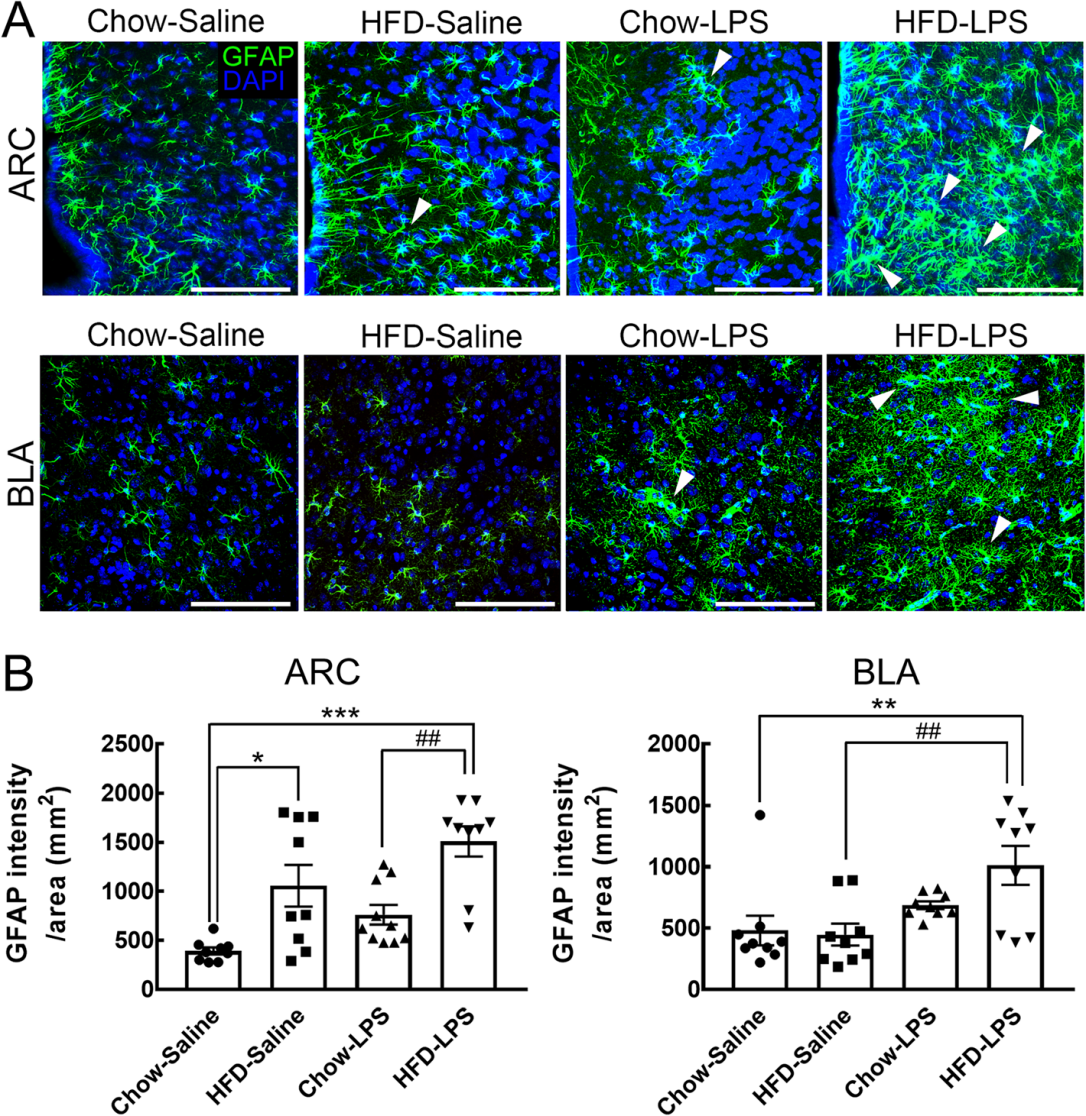
Enhanced astrocyte morphogenesis was observed in the ARC and BLA of mice intermittently administered by LPS and chronically fed by HFD. **a** Brain tissue sections containing the ARC and BLA were prepared from animals from the four groups after feeding for 5 months (Fig. 1a) and then subjected to GFAP immunofluorescence (green) and DAPI nuclear counterstaining (blue). Representative astrocytes with intense elaborated processes are indicated by arrowheads. **b** The immunoreactive intensity of GFAP in the ARC or BLA of animals from the four groups was quantified. In addition, the average cell body size of microglia in the two regions was measured. The data are presented as the mean ± SEM (*n* = 9 tissue sections from 3 animals from each group). *p < 0.05, **p < 0.01, ***p < 0.001 versus the chow-saline group; ^##^*p* < 0.01 versus the HFD-LPS group. Scale bar in *A* = 100 μm

Given that the complex elaboration of astrocytic processes reflects the multiple functions of astrocytes in the CNS [26, 27], the morphology of astrocytes in distinct brain regions of animals from the four groups was examined at 5 months using immunofluorescence for the astrocytic cytoskeleton protein GFAP (Fig. 1). The results showed that early LPS administration combined with chronic HFD feeding enhanced the complexity of astrocytic processes and GFAP intensity in the ARC when compared to those observed in both the chow-saline and chow-LPS groups (Fig. 8a, arrowheads; Fig. 8b). Chronic HFD feeding for 5 months also increased GFAP expression in ARC astrocytes compared to that in the chow-saline group (Fig. 8b). Note that an insignificant difference in GFAP intensity was found between the chow-saline and chow-LPS groups.

Interestingly, early LPS administration induced the elaboration of astrocytic processes and caused an increase in GFAP expression in the BLA of chow-fed mice (Fig. 8a, arrowheads; Fig. 8b) while chronic HFD feeding did not affect the expression of GFAP in the BLA at 5 months (Fig. 8b, d). The expression of GFAP in the BLA, like the elaboration of astrocytic processes (Fig. 8a, arrowheads), was much higher in the HFD-LPS group than in both the chow-saline and HFD-saline groups (Fig. 8b). However, no  differences in astrocytic morphology was observed  in the NAc, ACC, and insula between the four animal groups (Fig. S2). Yet, astrocytes in the insula displayed elaborate cell processes after chronic HFD feeding for 5 months (Fig. S4, arrowheads). The finding that the elaboration of astrocytic processes in the ARC and BLA was enhanced by early LPS administration and chronic HFD feeding indicates that early LPS exposure resulted in the susceptibility of astrocytes in the two brain regions to continuous exposure to HFD feeding.

## Discussion

This study shows that intermittent peripheral administration of LPS at early time points induces extensive exploratory behavior 5 months after exposure to chow, whereas this behavior is suppressed by chronic HFD feeding. Our results demonstrate that early LPS administration might prime microglia in the ARC, NAc, and insula, causing microglia to alter their response to chronic HFD feeding. However, chronic HFD feeding might modulate the response of neural cells to suppress the stimulation of exploratory behavior by early LPS administration. Our findings also reveal that astrocyte morphogenesis is induced by the combination of early repeated LPS administration and chronic HFD feeding and that intermittent challenges with LPS at an earlier time might induce glial priming and change the sensitivity of glial cells to subsequent inflammatory stimulation by chronic HFD feeding.

An i.p. bolus injection of LPS has been shown to induce systemic peripheral inflammation at the acute stage, maintain a high level of TNF-α protein in the brain for up to 10 months, and delay the death of dopaminergic neurons [28]. Findings from other studies have also shown that the peripheral administration of LPS can maintain microglia activation in sensitive brain regions through either neuronal or humoral signaling pathways [9, 29, 30]. Moreover, microglia with hypertrophic shapes were persistently observed in the BLA, ACC, and insula of the chow-LPS group at 5 months, suggesting that the three intermittent injections of LPS at early time points might trigger long-term influences on the microenvironment in these brain regions. HFD feeding for 5 months also induced a long-term effect on the activation of microglia in the ARC, NAc, ACC, and insula. Nevertheless, endotoxin tolerance following repeated injections of LPS is a concern [31]. In our study, LPS was noncontinuously administered three times within 2 months during feeding. In addition, although our observations showed that significant increases in the hypothalamic gene expression and plasma levels of TNF-α and IL-1β were only detected 24 h after the first injection of LPS, activated microglia were detected in the hypothalamus of chow-fed mice 24 h after each LPS injection (Fig. S1). Thus, the results indicating the inhibition of microglial activation in the HFD-LPS group suggest that LPS administration at early time points can trigger alterations in microglia and immune cell responses to subsequent stimulation by chronic HFD feeding. This possibility is further supported by the finding that the activation of microglia in the affected brain regions after either peripheral LPS injections or chronic HFD feeding was significantly suppressed by the combinatorial effect of early LPS administration and chronic HFD feeding. Based on the findings that repeated peripheral immune challenges with LPS or cytokines for consecutive days can induce innate immune memory in microglia [20], it is likely that microglial immune memory might be induced by challenges with intermittent LPS injections and daily HFD feeding. It remains to be further determined how the two inflammatory stimuli interact and subsequently mediate microglial activity.

Hypothalamic microglial activation is considered an important regulator of HFD-induced obesity [32]. Our finding that the body weight of obese mice was not affected by the suppression of microglial activation in the ARC in the HFD-LPS group suggests that the inhibition of microglial activation in the ARC might not be sufficient to reduce the body weight of obese mice after chronic feeding by HFD. Hypothalamic astrogliosis occurs in obese mice and is eliminated after a high nutrient diet is replaced by a chow diet [33]. A recent study also indicated that acute intense weight loss is able to induce astrocyte gliosis in the ARC of the hypothalamus [34]. Although body weight loss or gain was not observed in HFD-fed mice that received early LPS administration, ARC astrocytes in the HFD-LPS group displayed highly branched cell processes compared to those in the HFD-saline group. Accordingly, in our study, the change in the morphogenesis of ARC astrocytes was possibly due to the response of these astrocytes to alterations in the microenvironment and microglia priming induced by early LPS administration in obese mice. The BLA is an important structure in the amygdala that is the key brain region in the network of anxiety-related information processing [35, 36]. The finding that microglial activation in the BLA was induced in chow-fed mice that received early LPS administration but not in HFD-fed mice reveals that BLA microglia are more susceptible to early LPS administration than chronic HFD feeding.

Since open-arm exploration by stressed rodents is increased after exposure to anxiolytic drugs [37, 38], our finding that early LPS administration increased open-arm exploratory behavior in chow-fed mice suggests that intermittent LPS injections might induce anxiolytic effects in mice. In addition, increased exploration in the open arm or the center of the open field can be interpreted as a tendency toward the development of novel/risk-taking behavior [39]. Thus, our behavior study demonstrates that chronic HFD feeding might attenuate anxiolytic-like and risk-taking exploratory behavior in mice exposed to early LPS administration. Nevertheless, to corroborate the clinic potential of HFD intake to treat this behavioral abnormality, the effective components in HFD should be further characterized. The sex differences in the occurrence of depression and anxiety after a single injection with LPS into rodents have been documented [40]. It would be interesting to further determine the effect of the repeated LPS administrations on the affective behavioral development in female mice. Overall, this is the first study showing that intermittent peripheral challenges with LPS can induce the development of atypical exploratory behavior in male mice.

The findings of our previous study and other studies indicate that microglial activation-associated inflammation in the amygdala is involved in the development of fear and anxious behaviors [23, 41, 42]. However, in this study, we observed that the reduction in LPS-induced microglial activation in the BLA of the mice receiving chronic HFD feeding was correlated with reduced LPS-triggered explorative behavior/risk-taking behaviors in HFD-fed mice. Through the examination of brain-derived neurotrophic factor (BDNF) that has been considered as the critical regulator associated with stress, fear, and anxiety [43], a change in amygdalar BDNF levels in the HFD-saline and chow-LPS groups was not evident, although its level was significantly reduced in the ACC of the three animal groups (HFD-saline, chow-LPS, and HFD-LPS) compared to that observed in the control group (Fig. S5). In addition that the critical factor(s) involved in the development of LPS-induced unusual behavior need to be characterized, the following issues remain to be resolved. First, were BLA microglia involved in the processing of exploratory behavior induced by early LPS administration in chow-fed and HFD-fed mice? Second, was the decline in LPS-induced microglial activation in the BLA due to the alteration of the microenvironment in the BLA by neural pathway(s) triggered by chronic HFD feeding? Furthermore, our GFAP immunofluorescence data in distinct brain regions reveal that astrocytes in the BLA, ACC, and insula are more sensitive to intermittent LPS injections than those analyzed in the ARC. However, chronic HFD feeding might trigger an undefined signaling pathway to increase BLA astrocyte morphogenesis in mice that receive early LPS administration. However, although the role of astrocytes with intense elaborated processes in LPS-induced exploratory behavior remains to be uncovered, astrocytes might sense altered microenvironments in the examined brain regions under the influence of early LPS administration and chronic HFD feeding to undergo morphogenesis. Nevertheless, the mechanisms of the interplay between the effect of distinct peripheral immune stimuli on glial activity in the CNS and animal behavior remain to be uncovered.

## Conclusions

In summary, our findings demonstrate that intermittent peripheral immune challenges before and after significant body weight increases in HFD-fed mice can prime microglia in the ARC and other brain regions to amend microglial response to chronic HFD feeding, which might further reduce the effect of LPS on the development of exploratory behavior.

## Supplementary information

**Additional file 1: Figure S1**. Morphological alteration of microglia in the hypothalamic arcuate nucleus was induced at 24 h after each LPS administration. Left panel: brain tissue sections containing the hypothalamic arcuate nucleus (ARC) were prepared from animals from the four groups at 24 h after each injection (1W, 2W, and 8W) with saline or LPS, and then subjected to Iba1 immunofluorescence (red) and DAPI nuclear counterstaining (blue). Right panel: the number of Iba1+ microglia accumulated in the ARC (per mm^2^) in the four groups was quantified. The data are presented as the mean ± SEM (n = 9 tissue sections from 3 animals from each group). *p<0.05, **p<0.01, ***p< 0.001 versus Chow-Saline. Scale bar in A = 50 μm. **Figure S2.** Examination of the food intake of Chow- or HFD-fed mice receiving Saline or LPS injections. The food intake of the four animal groups (Chow-Saline, HFD-Saline, Chow-LPS, HFD-LPS) was measured weekly for 5 months. The data are presented as the mean ± SEM (n = 6 animals in each group). ***p<0.001 HFD-Saline versus Chow-Saline. ###*p*<0.001 HFD-LPS versus Chow-LPS. $ *p*<0.05, $$$*p*<0.001 Chow-LPS versus Chow-Saline. **Figure S3.** Examination of animal walking in the close arm. After the animals in the four groups were fed by Chow or HFD up to 5 months, and then subjected for EPM assay. Their behaviors in the open arms are shown in Fig. 4. In addition, time (seconds) spent in the close arm and entries into the close arms, and total distance were measured. We noticed that no difference in time spent and entry number in the close arm was detected in the four groups. **Figure S4.** Intense GFAP immunoreactivity detected in insula of HFD-fed mice. The brain tissue sections containing NAc, ACC, or insula were prepared from the four animal groups after the feeding for 5 months (Fig. 1), and then subjected to GFAP immunofluorescence (green) with DAPI nuclear counterstaining (blue). The representative astrocytes with intense elaborated processes are indicated by arrowheads in ACC (Chow-LPS) and insula (HFD-Saline, and HFD-LPS). Note that low GFAP immunoreactivity was observed in NAc of the four animal groups. Scale bar in A = 100 μm. **Figure S5.** Examination of BDNF protein levels in ACC and amygdala at 5 month after HFD feeding. Tissues containing ACC and amygdala were prepared from animals from the four groups after feeding for 5 months and then subjected to Western Blotting analysis using anti-BDNF antibody that can recognize pro-BDNF and mature BDNF. GAPDH level is referred as the loading control. The intensity of the bands corresponding to pro-BDNF (34 kDa), mature BDNF (14 kDa), and GAPDH was quantified. The data are presented as the mean ± SEM (n = 3 animals for Chow-Saline and HFD-Saline; n = 4 animals for Chow-LPS and HFD-LPS). ***p< 0.001 versus Chow-Saline; #*p*<0.05 versus Chow-LPS.

## Data Availability

Not applicable

## Abbreviations

ACC: Anterior cingulate cortex
ARC: Arcuate nucleus
BDNF: Brain-derived neurotrophic factor
BLA: Basolateral amygdala
CNS: Central nervous system
CyPA: Cyclophilin A
ELISA: Enzyme-linked immunosorbent assay
EPM: Elevated plus-maze
GFAP: Glial fibrillary acidic protein
HFD: High-fat diet
IL-1β: Interleukin-1β
i.p.: Intraperitoneal
LPS: Lipopolysaccharide
NAc: Nucleus accumbens
NIH: National Institutes of Health
OFT: Open field test
TNF-α: Tumor necrosis factor-α
