# Supplementary material for: Intermittent peripheral exposure to lipopolysaccharide induces exploratory behavior in mice and regulates brain glial activity in obese mice
Source: J Neuroinflammation. 2020 May 25;17:163. doi: 10.1186/s12974-020-01837-x (PMC7249324; doi:10.1186/s12974-020-01837-x)
Supplement: Supplementary file 1 — Additional file 1: Figure S1. Morphological alteration of microglia in the hypothalamic arcuate nucleus was induced at 24 h after each LPS administration. Left panel: brain tissue sections containing the hypothalamic arcuate nucleus (ARC) were prepared from animals from the four groups at 24 h after each injection (1W, 2W, and 8W) with saline or LPS, and then subjected to Iba1 immunofluorescence (red) and DAPI nuclear counterstaining (blue). Right panel: the number of Iba1+ microglia accumulated in the ARC (per mm2) in the four groups was quantified. The data are presented as the mean ± SEM (n = 9 tissue sections from 3 animals from each group). *p<0.05, **p<0.01, ***p< 0.001 versus Chow-Saline. Scale bar in A = 50 μm. Figure S2. Examination of the food intake of Chow- or HFD-fed mice receiving Saline or LPS injections. The food intake of the four animal groups (Chow-Saline, HFD-Saline, Chow-LPS, HFD-LPS) was measured weekly for 5 months. The data are presented as the mean ± SEM (n = 6 animals in each group). ***p<0.001 HFD-Saline versus Chow-Saline. ###p<0.001 HFD-LPS versus Chow-LPS. $ p<0.05, $$$p<0.001 Chow-LPS versus Chow-Saline. Figure S3. Examination of animal walking in the close arm. After the animals in the four groups were fed by Chow or HFD up to 5 months, and then subjected for EPM assay. Their behaviors in the open arms are shown in Fig. 4. In addition, time (seconds) spent in the close arm and entries into the close arms, and total distance were measured. We noticed that no difference in time spent and entry number in the close arm was detected in the four groups. Figure S4. Intense GFAP immunoreactivity detected in insula of HFD-fed mice. The brain tissue sections containing NAc, ACC, or insula were prepared from the four animal groups after the feeding for 5 months (Fig. 1), and then subjected to GFAP immunofluorescence (green) with DAPI nuclear counterstaining (blue). The representative astrocytes with intense elaborated processes are indicated by [file 12974_2020_1837_MOESM1_ESM.doc]

**
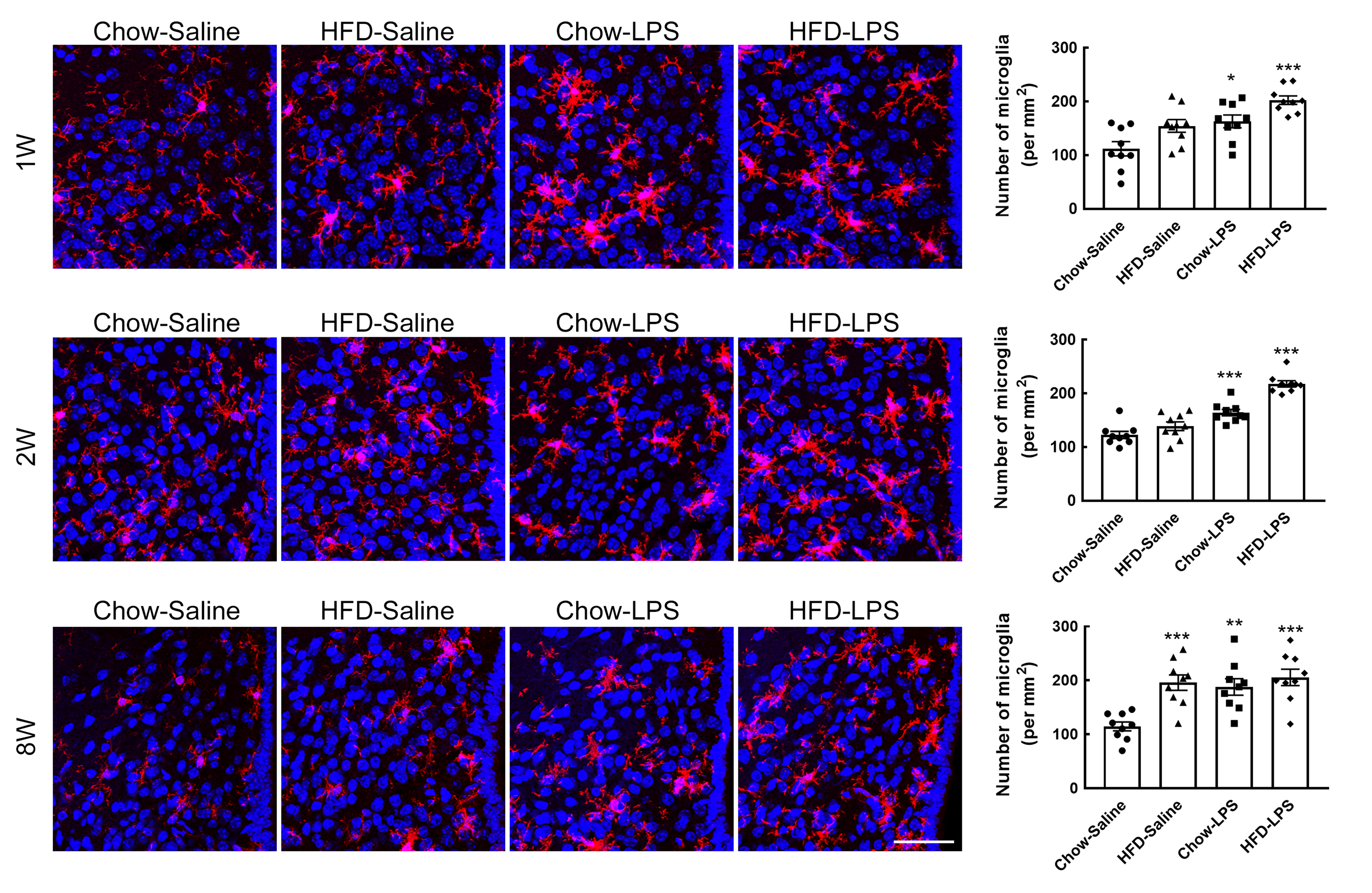
**

**Figure S1.** Morphological alteration of microglia in the hypothalamic arcuate nucleus was induced at 24 h after each LPS administration. Left panel: brain tissue sections containing the hypothalamic arcuate nucleus (ARC) were prepared from animals from the four groups at 24 h after each injection (1W, 2W, and 8W) with saline or LPS, and then subjected to Iba1 immunofluorescence (red) and DAPI nuclear counterstaining (blue). Right panel: the number of Iba1+ microglia accumulated in the ARC (per mm^2^) in the four groups was quantified. The data are presented as the mean ± SEM (n = 9 tissue sections from 3 animals from each group). **p*<0.05, ***p*<0.01, ****p*< 0.001 versus Chow-Saline. Scale bar in A = 50 μm.


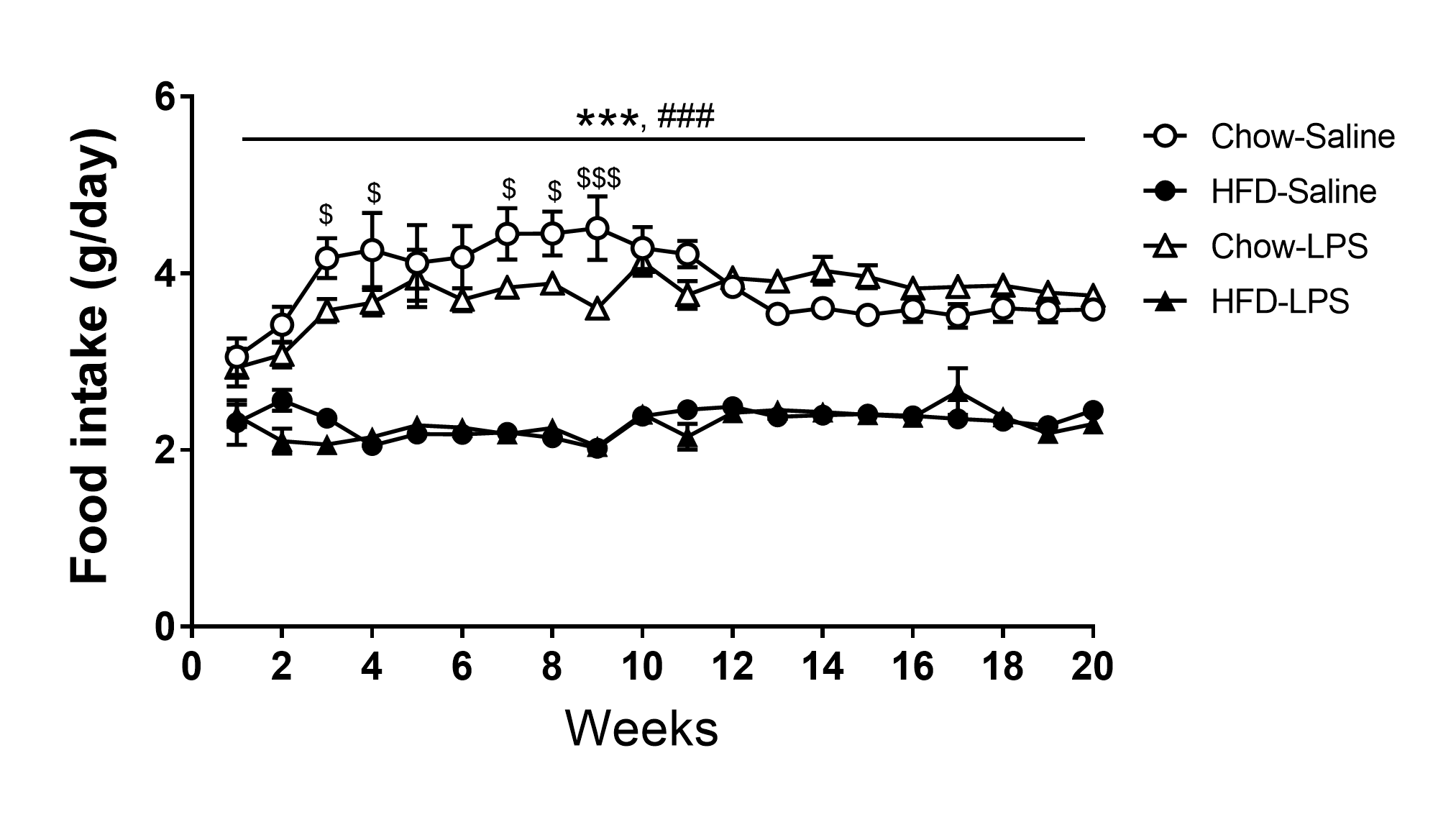


**Figure S2.** Examination of the food intake of Chow- or HFD-fed mice receiving Saline or LPS injections. The food intake of the four animal groups (Chow-Saline, HFD-Saline, Chow-LPS, HFD-LPS) was measured weekly for 5 months. The data are presented as the mean ± SEM (n = 6 animals in each group). ****p*<0.001 HFD-Saline versus Chow-Saline. ###*p*<0.001 HFD-LPS versus Chow-LPS. $ *p*<0.05, $$$*p*<0.001 Chow-LPS versus Chow-Saline.


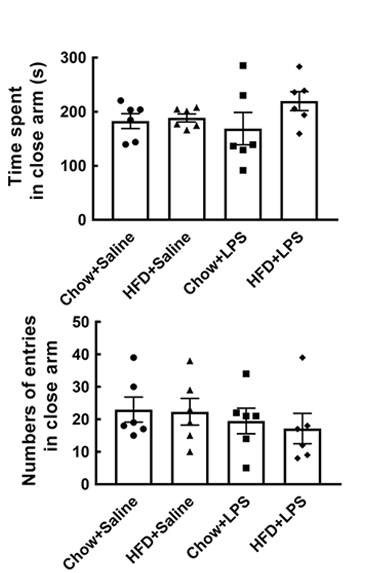


**Figure S3.** Examination of animal walking in the close arm. After the animals in the four groups were fed by Chow or HFD up to 5 months, and then subjected for EPM assay. Their behaviors in the open arms are shown in Fig. 4. In addition, time (seconds) spent in the close arm and entries into the close arms, and total distance were measured. We noticed that no difference in time spent and entry number in the close arm was detected in the four groups.


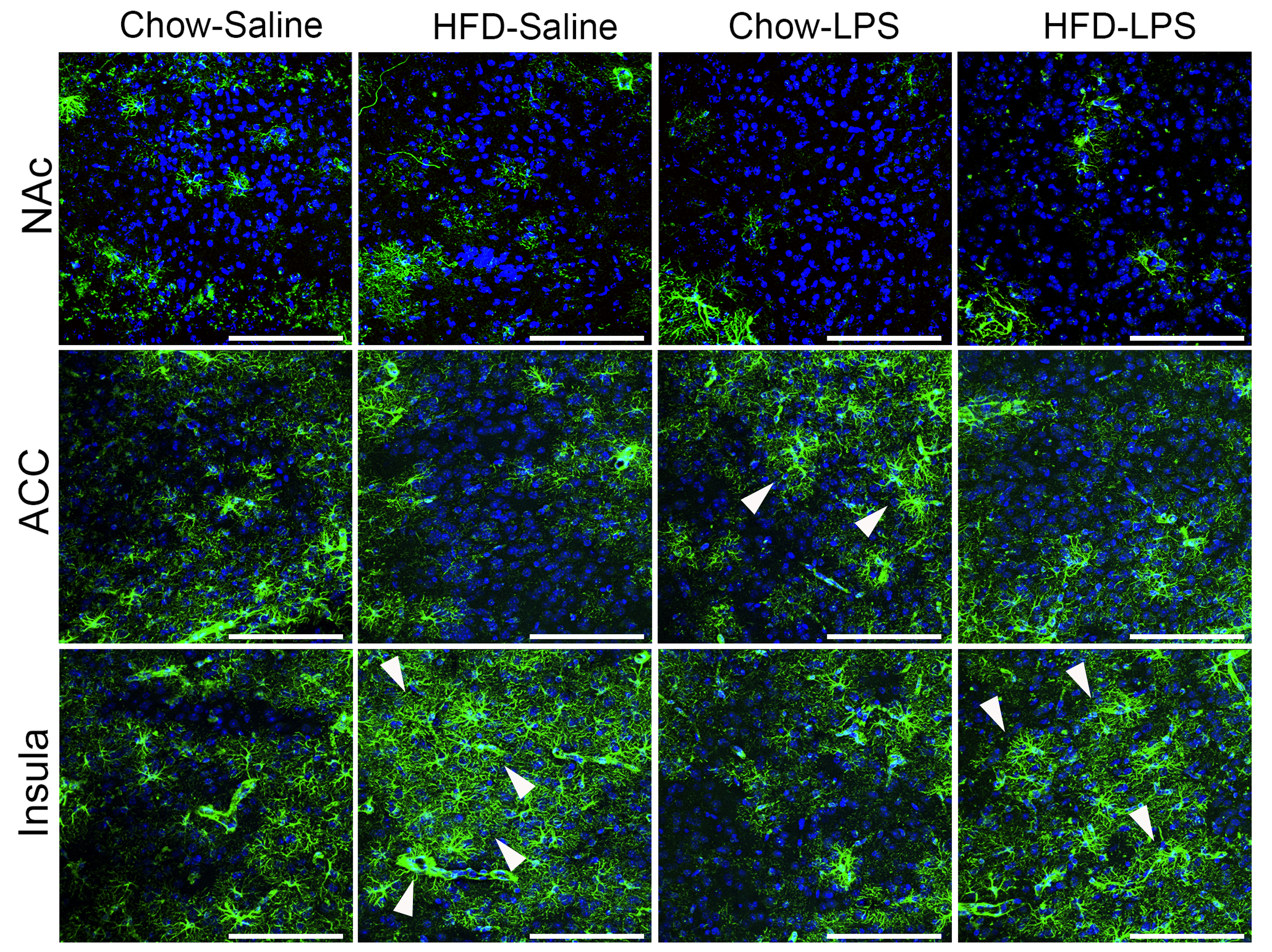


**Figure S4.** Intense GFAP immunoreactivity detected in insula of HFD-fed mice. The brain tissue sections containing NAc, ACC, or insula were prepared from the four animal groups after the feeding for 5 months (Fig. 1), and then subjected to GFAP immunofluorescence (green) with DAPI nuclear counterstaining (blue). The representative astrocytes with intense elaborated processes are indicated by arrowheads in ACC (Chow-LPS) and insula (HFD-Saline, and HFD-LPS). Note that low GFAP immunoreactivity was observed in NAc of the four animal groups. Scale bar in A = 100 μm.


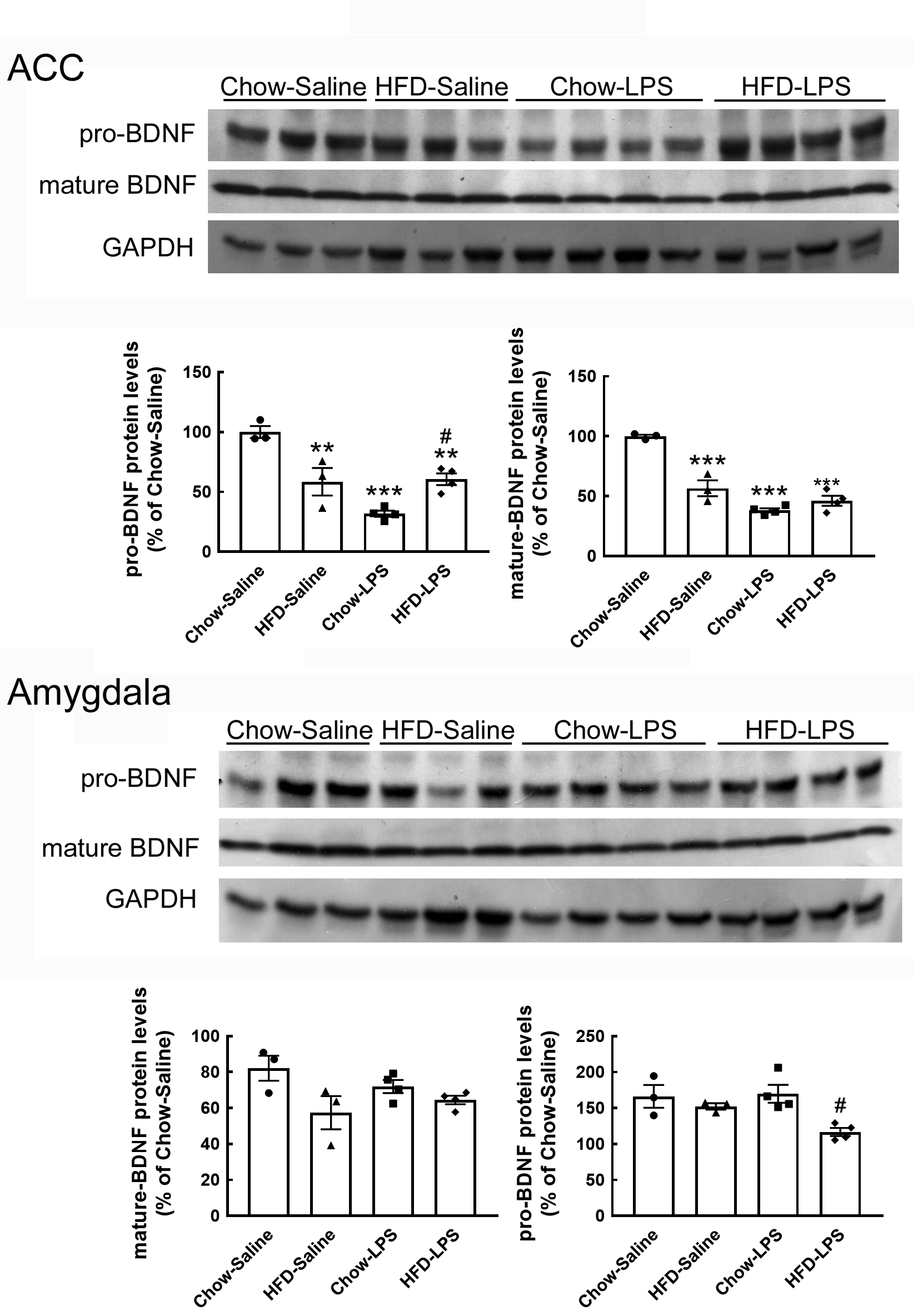


**Figure S5.** Examination of BDNF protein levels in ACC and amygdala at 5 month after HFD feeding. Tissues containing ACC and amygdala were prepared from animals from the four groups after feeding for 5 months and then subjected to Western Blotting analysis using anti-BDNF antibody that can recognize pro-BDNF and mature BDNF. GAPDH level is referred as the loading control. The intensity of the bands corresponding to pro-BDNF (34 kDa), mature BDNF (14 kDa), and GAPDH was quantified. The data are presented as the mean ± SEM (n = 3 animals for Chow-Saline and HFD-Saline; n = 4 animals for Chow-LPS and HFD-LPS). ****p*< 0.001 versus Chow-Saline; #*p*<0.05 versus Chow-LPS.
